# The Horizon of a Therapy for Rare Genetic Diseases: A “Druggable” Future for Fibrodysplasia Ossificans Progressiva

**DOI:** 10.3390/ijms19040989

**Published:** 2018-03-26

**Authors:** Serena Cappato, Francesca Giacopelli, Roberto Ravazzolo, Renata Bocciardi

**Affiliations:** 1 Department of Neurosciences, Rehabilitation, Ophthalmology, Genetics, Maternal and Child Health, University of Genoa, 16132 Genoa, Italy; serena.cappato@edu.unige.it (S.C.); francesca.giacopelli@unige.it (F.G.); rravazzo@unige.it (R.R.); 2 UOC Genetica Medica, IRCCS Istituto Giannina Gaslini, 16147 Genoa, Italy

**Keywords:** fibrodysplasia ossificans progressiva (FOP), bone morphogenetic proteins (BMPs), Activin A, high-throughput screening, drug discovery, drug repositioning

## Abstract

Fibrodysplasia ossificans progressiva (FOP) is a rare genetic condition characterized by progressive extra-skeletal ossification leading to cumulative and severe disability. FOP has an extremely variable and episodic course and can be induced by trauma, infections, iatrogenic harms, immunization or can occur in an unpredictable way, without any recognizable trigger. The causative gene is *ACVR1*, encoding the Alk-2 type I receptor for bone morphogenetic proteins (BMPs). The signaling is initiated by BMP binding to a receptor complex consisting of type I and II molecules and can proceed into the cell through two main pathways, a canonical, SMAD-dependent signaling and a p38-mediated cascade. Most FOP patients carry the recurrent R206H substitution in the receptor Glycine-Serine rich (GS) domain, whereas a few other mutations are responsible for a limited number of cases. Mutations cause a dysregulation of the downstream BMP-dependent pathway and make mutated ACVR1 responsive to a non-canonical ligand, Activin A. There is no etiologic treatment for FOP. However, many efforts are currently ongoing to find specific therapies targeting the receptor activity and the downstream aberrant pathway at different levels or targeting cellular components and/or processes that are important in modifying the local environment leading to bone neo-formation.

## 1. Fibrodysplasia Ossificans Progressiva (FOP) and the *ACVR1* Gene

Fibrodysplasia ossificans progressiva (FOP, OMIM 135100) is a rare genetic condition affecting 1:2,000,000 people. Its clinical presentation is characterized by the presence of a peculiar congenital malformation of the great toes and progressive extra-skeletal ossification (heterotopic ossification, HO) leading to cumulative and severe disability. Other skeletal malformations and signs may be variably associated with the phenotype, such as the fusion of cervical *vertebrae*, thumb malformation, presence of osteochondromas, etc. [1,2]. FOP has an extremely variable and episodic course: acute phases culminating in bone neo-formation affecting skeletal muscles, tendons, ligaments, joints alternate with quiescent phases. FOP flare-ups can be induced by trauma, infections, medical and surgical procedures, immunizations or can occur in an unpredictable way, without any recognizable trigger.

The gene mutated in FOP patients is *ACVR1* (OMIM 102576), also known as *Alk-2*, encoding a type I, trans-membrane receptor for bone morphogenetic proteins (BMPs) belonging to the TGF-β superfamily [3]. The receptor has a serine-threonine kinase activity associated with the cytoplasmic domain of the protein.

In the context of the TGF-β superfamily, three type I receptors (Alk-2/-3/-6) and the BMPR2 type II receptor act as BMP receptors. The signaling is initiated by BMP interaction with an heteromeric receptor complex consisting of type I and II molecules—type II receptors activate type I, by transphosphorylating their GS-domain. Receptor stoichiometry and ligand-binding dynamics contribute to the tuning and specificity of the downstream signaling, which can proceed into the cell through two main pathways, a canonical, SMAD1/5/8-dependent signaling and a p38-mediated cascade [4].

The GS domain is specific for type I BMP receptors, is highly conserved and plays an important role in regulating receptor function. The GS domain binds the inhibitor FKBP12 protein, keeping the receptor in an inactive conformation in the absence of ligands or in presence of sub-optimal concentrations, thus blocking leaky signaling into the cell. Upon interaction with ligands, FKBP12 is released and the GS domain can be phosphorylated by type II receptors, thus activating the intra-cellular cascade [5].

The vast majority of FOP patients carry a recurrent nucleotide transition (c.617G>A) in the fourth coding exon of the *ACVR1* gene, causing the substitution of a conserved residue in the GS domain of the protein, the R206H. A few other mutations affecting other residues in the GS or affecting the kinase domain are responsible for a limited number of cases (Table 1) [1,2,3,6,7].

Mutations cause a dysregulation of the downstream BMP-dependent pathway and make mutated *ACVR1* responsive to a non-canonical, and usually non osteogenic ligand, Activin A (ActA) [8,9].

Nevertheless, the presence of the mutation does not seem to be sufficient to cause HO. The formation of the ectopic bone in FOP is a complex and multi-step process combining an inciting event, signals arising from the site of injury released by damaged cells and/or from cells of the inflammatory component, and presence of progenitor cells able to differentiate into chondroblasts and/or osteoblasts in the appropriate inductive environment. Many different cell types, all carrying the causative mutation, participate in this process.

So far, no etiologic treatment is available to cure FOP, preventing or blocking flare-ups and thus avoiding progressive and severe disability culminating in the entrapment of patients in a second skeleton of ectopic bone. Patients are usually treated with corticosteroids, prevention of trauma is strongly recommended and unnecessary medical/surgical procedures have to be carefully avoided (for comprehensive guidelines, see [10]).

However, many efforts are currently ongoing to find specific therapies targeting the receptor activity and the downstream aberrant pathway at different levels, or targeting cellular components and/or processes that are important in modifying the local environment conducive to bone neo-formation. The identification of *ACVR1* as the gene responsible for FOP and the studies aimed at investigating the molecular and cellular processes taking place in FOP pathophysiology have provided indications of several druggable targets suitable for the development of new therapeutic approaches for FOP. This review is aimed at providing an overview of these studies and of the most recent approaches to developing new therapeutic strategies.

## 2. Translating the Results of Basic Research into the Development of New Therapeutic Strategies

### 2.1. Targeting the Altered Signaling

#### 2.1.1. FOP Mutations and BMP Signaling

Due to its importance in bone biology and homeostasis, the BMP pathway was already considered a candidate for FOP pathogenesis even before the discovery of *ACVR1* as the responsible gene. A few years before this discovery, in vivo experiments showed that implants of BMP-embedded matrigel in mouse skeletal muscles induced the formation of ectopic ossicles through a cartilage intermediate, after an early phase of tissue damage and inflammation [11,12]. Similarly, ectopic bone formation was also described in transgenic mice overexpressing BMP4 under the control of the NSE promoter [13], indicating that hyper-activation of this signaling may subvert the normal tissue repair process promoting ectopic bone formation.

Studies performed on peripheral blood lymphocytes from FOP patients showed that the expression of BMPRIA was increased compared to controls, and that these cells had an altered response to BMP4 treatment with upregulation of p38-MAPK kinase signaling compared to cells from non-FOP individuals. The altered signaling was also observed for the canonical SMAD-mediated pathway and confirmed by the increase in *ID1* and *ID3* expression, known BMP target genes [14,15].

The discovery of *ACVR1* as the gene responsible for FOP confirmed this hypothesis and protein modeling of the recurrent R206H mutation predicted an increase of the function effect on the receptor activity [3]. Most importantly, the identification of the gene defect allowed the performance of functional characterizations of the mutant protein. In vitro assays demonstrated that the R206H substitution conferred to the receptor a ligand-independent activity, with increased SMAD1/5-mediated signaling, which was also observed after the induction of muscle injury in mice [16]. This was considered of particular interest for FOP, in which ectopic bone formation can be triggered by muscle trauma.

The functional consequences of the R206H mutation were further investigated in different cellular models, thus confirming the gain-of-function effect in the absence of ligand, a decreased affinity of the mutated receptor for the binding of the negative modulator FKPB12, and enhanced chondrogenesis in chick limb micromass cultures [17]. Moreover, the effect was also tested in vivo in zebrafish embryos. The BMP pathway is highly conserved and *Alk-8* is the zebrafish functional ortholog of the human *Alk-2*. This pathway is involved in the determination of the dorso-ventral axis of the embryo. In the absence of BMP signaling due to the genetic inactivation of *Alk-8*, the embryo shows a dorsalization phenotype particularly evident in the tail. The injection of the active R206H mutant partially rescued this phenotype, while injection in wild-type embryos led to a ventralized phenotype associated to hyper-functional BMP signaling [17].

Only very few FOP patients carry an *ACVR1* mutation different from the recurrent R206H (Table 1). These rare mutations affect other conserved residues of the GS domain or map into the kinase domain of the protein. A gain-of-function effect was also predicted for these substitutions. Indeed, ligand-independent signaling, increased SMAD1/5 activation, enhanced chondrogenic and osteogenic properties in different cellular models, and a reduction in FKBP12 binding were also observed for the L196P, Q207E (GS domain) and G356D (kinase domain) substitutions [18,19,20,21]. Moreover, in vivo studies in zebrafish embryos demonstrated that G356D/W/R substitutions also enhanced the SMAD1/5 signaling and induced embryo ventralization with loss of dorsal cell fate markers, as observed for the R206H mutation [22]. The activation state of the different mutants is not exactly the same but they are still BMP-responsive and maintain the ability to be down-regulated by known BMP inhibitors such as regulatory SMAD6 and 7, over-expression of FKBP12 or pharmacological treatment with known inhibitors of the signaling such as dorsomorphin and LDN193189 [18,20,21].

Dorsomorphin was identified by screening a chemical library of small molecules that dorsalize zebrafish embryos, and was found to block BMP signaling by inhibiting type I receptors Alk-2, Alk-3 and Alk-6 [23]. Reiterative synthesis and testing of specific derivatives of the parent molecule aimed at improving the potency, pharmacokinetics and specificity towards the Alk-2 receptor, while trying to limit off-target effects, led to the identification of LDN-193189, DMH1, and LDN-212854 analogues [24,25,26,27]. These molecules act as inhibitors of the receptor kinase function by interacting with the ATP-binding pocket of the kinase domain, thus competing with ATP and keeping the receptor in an inactive conformation.

The conditional caAlk2^Q207D^ mouse model was used for evaluating in vivo the effect of treatment with LDN-193189. This is a Cre-dependent mouse model expressing the human *ACVR1/Alk-2* cDNA carrying the engineered Q207D substitution of the GS domain (caALK2^Q207D^ mouse model) [28]. This residue is highly conserved among type I receptors and was previously characterized from a functional point of view by mutational analysis of the phosphorylation sites in the TGF-β1 receptor GS domain [29]. The mutation caused a constitutive activation of the receptor, and the same effect was also observed when inserted in the *ACVR1* cDNA [23,28,29].

The baseline phosphorylation level of SMAD1/5/8 in cells derived from these mice was increased after the induction of transgene expression, indicating a constitutive signaling mediated by the mutated receptor. These cells were also hyper-responsive to the presence of BMP ligands and treatment with LDN-193189 efficiently inhibited the dysregulated signaling. The same beneficial effect was also demonstrated in vivo. These mice developed HO lesions after local induction of the transgene expression by recombinase injection in limb muscles. Treatment of mice with LDN-193189 allowed the evaluation of the pharmacokinetics of the molecule and to demonstrate its ability in reducing the extent of ectopic bone formation [23].

The action of the above-mentioned inhibitors is linked to their interaction with a highly conserved region of the type I receptor kinase domain, therefore it is not restricted to Alk-2 function nor specific to its FOP-associated mutated form [23,24,26,27]. Moreover, the potency and the efficacy to block the formation of ectopic bone in vivo might be improved [23] and studies on the toxicity and tolerability of long-term treatment with these molecules should be carefully evaluated in further pre-clinical studies.

However, these works have opened the door to the targeting of the BMP dysregulated signaling in FOP by developing a pharmacological approach. Among the dorsomorphin derivatives developed, LDN-212854 is considered so far the molecule with the highest specificity for Alk-2 compared to other analogues of the same pharmacological class. Recently, the crystallographic structure of the molecule bound to the Alk2 kinase domain has been resolved and the Alk-2 residues involved in this interaction have been identified [30]. Careful analysis of these structures could disclose small conformational or sequence changes that might provide information regarding how to derive more potent and receptor-specific inhibitors [30], which might be further developed as a potential drug for FOP.

Therapeutic approaches based on the development of small molecules are being successfully applied to genetic diseases such as cystic fibrosis and other genetic conditions [31,32,33]. However, the development of a drug starting from a potentially beneficial but newly identified molecule is a very long and expensive path. Drug repositioning, i.e., the use of known drugs and compounds to treat diseases different from those for which they were originally employed, provides a significant advantage in term of time and cost, compared to the development of new molecules [33].

Recently, Yamamoto and coworkers [34] applied a drug repositioning approach by screening a collection of FDA-approved drugs for their ability to interfere with the signaling mediated by the R206H mutated *ACVR1* transfected in C2C12 cells. They found that two antianginal agents, fendiline hydrochloride and perhexiline maleate, efficiently suppressed the altered signaling. Both drugs were then tested in vivo in a BMP-induced mouse model of ectopic ossification showing a good reduction in the volume of the newly-formed ossicles in animals treated with perhexiline [34]. As this drug is commonly and safely used in the prophylactic treatment of angina, an open-label clinical trial was performed by administrating perhexiline to five FOP patients in order to evaluate the efficacy of this molecule in the management of the disease [35]. Three patients were in a quiescent phase of the disease during the entire period of the study and the remaining two experienced flare-ups with a worsening of their clinical condition. Therefore, although the drug was well tolerated, the study did not allow them to draw any conclusion about the clinical utility, if any, of perhexiline [35].

#### 2.1.2. FOP and Neofunction of the Mutated Receptor

In 2015, two seminal works achieved a critical step forward in the identification of pathogenic mechanisms responsible for FOP. Two independent research teams demonstrated that mutations associated with FOP conferred to the ACVR1 receptor the ability to bind and transduce the signal mediated by a non-canonical ligand, Activin A (ActA) [8,9]. ActA is a member of the TGF-β/BMP family of ligands. Binding to its cognate receptor molecules (type I, ACVR1B/Alk-4 and ACVR1C/Alk-7; type II: ACVR2A, ACVR2B and BMPR2) activates an intra-cellular cascade mediated by SMAD2/3 and modulates the expression of target genes after translocation into the nucleus. Usually ActA does not have osteogenic properties and is secreted by several cell types, among which cells of the immune system that play a fundamental role in the early phases of FOP lesions.

ActA can bind wild-type ACVR1 not only missing any signal transduction inside the cell but also exerting a negative effect of the BMP cascade [8,36]. On the contrary, binding of ActA to the R206H mutant receptor activates the downstream signaling through canonical SMAD1/5/8 mediators in several cell types expressing mutated cDNA, in induced pluripotent stem cells (iPSC) derived from FOP patients and in vivo, in mice models [8,9,36].

The acquired change in ligand specificity of the mutant receptor was shown to trigger endochondral ossification of patients’ induced mesenchymal stem cells (iMSC) cells derived from iPSC cells both in vitro and after the implantation of these cells in muscles of nude mice together with a source of ActA. Moreover, the receptor neo-function was also shown to alter the modulation of signaling operated by a negative feed-back loop involving the BMP antagonist GREM1, which is unable to counteract the cascade mediated by ActA/mutated ACVR1 [9].

To investigate the implication of these findings in vivo, Hatsell and coworkers [8] developed a physiologically relevant, genetically humanized conditional knock-in mouse model for FOP (Acvr1^[R206H]FlEx^). Upon pharmacological induction of *ACVR1*^R206H^ expression, mice spontaneously developed HO mimicking the FOP phenotype. In this mouse model, ectopic bone was also efficiently triggered by ActA injection, which was not observed in wild-type mice. Most importantly, formation of new HO was completely prevented by treatment with anti-ActA antibodies [8]. These data were also confirmed, more recently, in an independent mouse model, a conditional knock-in mouse in which the expression of the mutant Acvr1^R206H^ was induced by Cre-recombinase under the regulatory control of the endogenous *Acvr1* locus (Acvr1^tnR206H/+^) [36]. This work also allowed the authors to demonstrate the importance of the presence of the *ACVR1* wild-type allele able to bind ActA without triggering intracellular signaling, thus in part counteracting the effect of ActA on the mutated counterpart. Indeed, deletion of the wild-type allele in this mouse model led to an important increase in injury-mediated ectopic bone formation strictly dependent on Act A [36].

The complex of the above described results opened new directions in the knowledge of the molecular and cellular mechanisms responsible for FOP and provided the rationale for the application of anti-Activin-A reagents as new therapeutic tools, opening a path towards an etiologic treatment of FOP. A Phase 2 trial to verify the safety, tolerability and effects on abnormal bone formation of the use of an anti-ActA antibody (REGN2477) has been recently approved and is presently recruiting patients (ClinicalTrials.gov Identifier: NCT03188666) (Table 2).

On the other hand, these studies have also provided the basis to explore the pathways involved in HO formation initiated by ActA through mutated *ACVR1*, further allowing the identification of new druggable mechanisms.

Very recently, on these bases, a high-throughput screening (HTS) assay has been generated using FOP-patient-derived iPSCs (FOP-iPSCs) and applied to screen a library of small chemical compounds for their ability to interfere with the enhanced chondrogenesis exhibited by FOP-iPSCs upon ActA treatment [37]. This led to the identification of inhibitors of the mTOR pathway (rapamycin, everolimus, and temsirolimus) as effective modulators of the aberrant chondrogenesis typical of FOP-iPSCs. Transcriptome analysis of mesenchymal stem cells from FOP-iPSCs showed that mTOR-pathway-related genes were highly up-regulated compared to control cells upon treatment with ActA but not with BMPs or TGF-β ligands. Among these genes, one of the most differentially expressed in ActA treated cells was ENPP2, an enzyme that generates lysophosphatidic acid (LPA) a known activator of the mTOR signaling. The action of mTOR inhibitors on the differentiation of cells carrying the mutation does not involve the inhibition of the BMP cascade that is preserved, but suggests that mTOR signaling is a critical downstream pathway in enhanced chondrogenesis mediated by ActA/mutated ACVR1 and a possible new target to develop therapies [37]. In the same study, the authors described the suppression of HO formation by treatment with these molecules in vivo, in mice implanted with FOP-iPSCs cells together with a source of ActA [37], thus providing the basis for a possible repurposing of mTOR inhibitors in the management of FOP (see also section “Targeting the microenvironment of FOP local lesions” for results described by Kaplan et al. [38]).

## 3. Targeting Cell Progenitors and Differentiation Processes

The ectopic bone neo-formation in FOP is a complex and multi-step process. In a schematic view, an early inflammatory response precedes muscle degeneration, then local fibroproliferative reaction is followed by mesenchymal condensation, chondrogenesis and finally endochondral ossification. However, the exact origin and identity of progenitor cells undergoing the ectopic ossification process is still actively investigated and debated.

Immunohistochemical analysis of FOP lesions with markers specific for endothelial cells and lineage tracing experiments in mice have shown that cells of endothelial origin carrying mutated *ACVR1* may acquire a stem-like phenotype through an endothelial-to-mesenchymal transition. These cells would contribute, at least in part, not only to the vasculature of the ectopic bone but also to all the differentiation steps from early to mature lesions [12,39,40].

In a recent article, using the FOP mouse conditional knock-in mouse (Acvr1^[R206H]FlEx^, developed by Hatsell and coworkers [8]), the authors reported that different HO phenotypes are actually present in FOP. Ectopic ossification involving skeletal muscle is injury-dependent, whereas ossification of tendons, ligaments and joints seems to progress without any apparent trigger [41]. The authors systematically studied the origin of lesional precursor cells by activating the expression of the mutated *ACVR1* in a tissue-specific manner by breeding conditional Acvr1^[R206H]FlEx^ knock-in mice with mouse strains expressing Cre-recombinase under the control of a tissue-specific promoter. By these experiments, the authors concluded that progenitor cells contributing to injury-triggered versus “spontaneous” lesions were distinct and non-overlapping. *Mx*^+^ cells resident in the muscle *interstitium* are active in injury-induced muscle lesions, whereas *scleraxis*^+^ (*Scx^+^*) tendon progenitor cells are responsible for HO occurrence in tendons, ligaments and joints without any apparent trigger [41]. Moreover, these progenitor cells expressing the mutant ACVR1 seemed to robustly contribute to the ectopic cartilage but not to the latest stages of ectopic bone formation, for which also wild-type cells were recruited [41].

In independent studies, a source of potential lesional progenitor cells was also identified among multipotent mesenchymal cells resident in the skeletal muscle interstitium (Tie2^+^/PDGFRa^+^/Sca1^+^) and exhibiting adipogenic, chondrogenic, and osteogenic differentiation properties [42,43]. These cells, likely overlapping at least in part with those described by Dey and coworkers [41], were defined as fibro-adipogenic progenitors (FAPs) and found to robustly contribute to BMP-induced heterotopic ossification in mice, therefore representing a key cellular target for potential therapeutic intervention in HO treatment [42]. More recently, FAP cells were demonstrated to be the major source of progenitors for HO lesions by lineage tracing experiments in a conditional knock-in mouse in which the expression of the mutant Acvr1^R206H^ was induced by Cre-recombinase under the regulatory control of the endogenous Acvr1 locus (Acvr1^tnR206H^) [36]. Mutated FAPs were found to mediate both the injury-induced and the apparently spontaneous ectopic bone formation in all the anatomical districts described in FOP, and to contribute to both cartilage and bone formation, in apparent discrepancy with previous work by Dey et al. [41]. These processes were strictly ActA dependent, and treatment of these mice by a monoclonal anti-ActA antibodies was able to prevent HO formation for a long period even after a single administration [36].

Once activated and recruited, progenitor cells are pushed towards an extra-skeletal endochondral ossification process, mimicking the normal bone development in which a cartilage template is replaced by bone [44]. The process involves condensation, proliferation and differentiation of mesenchymal precursors into chondrocytes later replaced by osteoblasts leading to the formation of mature bone [45].

Studies of mesenchymal precursors from *Alk2^R206H^*^/+^ mice and from patients’ iPSC cells demonstrated that the mutated cells showed an enhanced chondrogenic potential in inductive conditions and this is in part due to the hyper-functioning BMP signaling [37,46,47]. This feature provides a robust contribution to the formation of HO [47] and makes chondrogenesis a critical step in the development of a mature and ossified lesion.

Chondrocyte differentiation and maintenance are complex processes involving different cellular pathways. Among these, signaling induced by retinoids certainly plays a pivotal role by maintaining condensing cells in a prechondrogenic, mesenchymal cell state, which prevents the differentiation into chondroblasts. Therefore, during normal development, chondroblast differentiation requires retinoid-receptor-mediated repression in order to proceed [48].

These observations from developmental biology studies provided hints to investigating the potential use of retinoid acid agonists in limiting/blocking both acquired and genetic HO typical of FOP, by preventing the formation of the cartilage scaffold [49].

Studies in a mouse model of BMP-induced ectopic ossification and in transgenic mice expressing constitutive-active caALK2^Q207D^ mutant showed that treatment with natural 13-*cis*-retinoic acid (13-*cis* RA) and even more, with synthetic, selective retinoic acid receptor γ (RARγ) agonists were able to inhibit HO formation by maintaining retinoid signaling active and thus blocking chondrogenic differentiation [50,51]. The final beneficial effect was also mediated by the concomitant down-regulation of the BMP-SMAD1/5/8 signaling operated by the RARγ agonists [51].

In this pharmacological class, Palovarotene appeared to be a suitable candidate for FOP, as it is a highly-specific RARγ agonist for which the safety and tolerability profile was already defined in a Phase 2 clinical trial for another condition [52]. The use of Palovarotene was demonstrated to be effective in reducing ectopic ossification formation in a BMP-induced HO mouse model both as a single treatment and in combination with corticosteroids [53]. A beneficial effect was also observed in a conditional-on knock-in mouse line carrying the recurrent, human ACVR1^R206H^ in which a significant reduction of HO formation and mitigation of the developmental defects linked to the mutated *ACVR1* were demonstrated [49,54].

These data provided the scientific basis to obtain the approval of the first interventional clinical trial to study the efficacy and safety of Palovarotene for the treatment of FOP (ClinicalTrials.gov Identifier: NCT03312634), presently on Phase 3 (Table 2).

## 4. Targeting the Expression of the Receptor

An approach aimed at down-modulating the hyper-active signaling in FOP might target the expression of the first switch of this cascade represented by the receptor itself. This could be achieved by down-modulating the expression of *ACVR1* both at the transcriptional and post-transcriptional level. This strategy implies that the molecular elements and mechanisms involved in controlling *ACVR1* gene expression have been known and understood at the best possible level.

To date, however, few studies have focused on this. Recently, the functional characterization of the genomic region located at the 5′ end of *ACVR1* has allowed the identification of a 2.9 kb sequence with promoter activity [55] and the design of a cell-based assay suitable for the screening of molecules potentially able down-modulate the expression of the gene at transcriptional level. This approach has been applied to the screening of a library of FDA-approved compounds and has allowed the identification of Dipyridamole, in use as platelet anti-aggregant, as a molecule able to decrease *ACVR1* expression, downstream signaling and to reduce ectopic bone formation in a BMP-induced mouse model of HO, suggesting a possible repurposing of the drug for FOP treatment, although further studies are needed to confirm its utility [56].

At the post-transcriptional level, few studies have demonstrated that expression of the *ACVR1* gene may be regulated by microRNAs. miR-365, miR-148b, miR-148a, miR-30-C and miR-130a have been shown to target the 3′UTR sequence of the *ACVR1* gene, thereby downregulating its expression [57,58,59,60,61]. MicroRNAs are considered a new class of diagnostic and prognostic biomarkers and they are regarded as emerging tools for therapeutic purposes for many conditions, and this might also apply to FOP in the future. However, critical issues about target specificity, safety and efficient delivery to the affected cells might be carefully addressed. For FOP, further studies aimed at clarifying these regulatory mechanisms and microRNAs involved are still needed. It would be important to investigate, not only the modulation of the *ACVR1* gene by microRNAs, but also that of other genes involved in the signaling cascade and in the differentiation processes responsible for ectopic bone formation, both in wild-type background and in mutated cells.

More recently, an alternative approach to decreasing the expression of the *ACVR1* gene has been based on the use of Anti-sense oligonucleotide (AON), able to bind a specific exon of the gene in the primary RNA, thus preventing the action of the splicing machinery and promoting the skipping of the selected exon. This strategy has been successfully applied to reframe the mutated dystrophin mRNA in skeletal muscle cells of Duchenne muscular dystrophy patients [62]. Introduction of AONs specific to the exon 8 of mouse *Acvr1* in C2C12 and endothelial cells has been shown to decrease the expression of the gene, probably by targeting the RNA with the skipped exon to the non-sense mediated degradation pathway and thus downregulating the BMP signaling [63].

However, the modulation of expression achieved by these strategies would affect both the wild-type and the mutated allele of the causative gene. From this point of view, RNA interference (RNAi) is considered a powerful tool to develop allele-specific therapies for genetic diseases.

Proof of principle that the RNAi approach might be successful in FOP has been obtained by the design of allele-specific RNAi molecules (ASP-RNAi) able to target the expression of mutant *ACVR1* alleles. The introduction of ASP-RNAi duplexes targeting the R206H or the G356D mutants in primary dental pulp cells from exfoliated deciduous teeth (SHED cells) or lymphoblasts from FOP patients has been proven to efficiently silence the mutated alleles and decrease the basal elevated BMP signaling to control levels [64,65].

The application of these approaches to cure genetic diseases such as FOP requires the identification of the best conditions to achieve an efficient systemic delivery of these molecules and appropriate cell targeting to obtain the required therapeutic effect. However, the results of these studies represent a proof-of-principle for the development of alternative therapeutic approaches.

## 5. Targeting the Immune System

The analysis of early FOP lesions, in biopsies from undiagnosed patients or obtained from mouse models of HO and FOP, has shown that the endochondral ossification process is preceded by a local, inflammatory reaction with tissue invasion by lymphocytes, mast cells and macrophages [66,67,68]. Local swelling is observed at the clinical onset of many acute phases and, although variable, response to early corticosteroid treatment can be obtained. The formation of ectopic bone in FOP is an episodic rather than a continuous process, with flare-ups often induced by inflammatory triggers following infections, trauma, immunizations, iatrogenic harms etc. (for a review, see [68]).

ActA, which plays an obligate role in FOP pathogenesis, is also extremely important for immune system biology [69,70] and immune cells can represent a source of this factor during the early inflammatory phase. Moreover, lesional progenitor cells might integrate other signals from the activated immune system also through the toll-like receptor (TLR) pathway that might provide fuel to further amplify the BMP signaling by positive cross-talk. This has been observed in stem cells from exfoliated deciduous teeth (SHED cells) from FOP patients, where inflammatory triggers activate the TLR3/TLR4 receptor, further stimulating the BMP signaling through the ECSIT adaptor, which links TLR signaling to the SMAD1/5/8 phosphorylation cascade [71]. Although these cells may not represent the precise source of ossifying progenitors responsible for ectopic bone formation in FOP, this study provides interesting data that may orient further studies.

All these observations have suggested the importance of an immune-mediated component in the FOP ossification process and different studies have tried to address in a systematic way this important issue. Experiments performed in a BMP-dependent mouse model of HO demonstrated that the ablation of macrophages markedly reduced the formation of ectopic bone, whereas targeting adaptive immune system cells caused a limitation of the lesions spreading [72]. This work confirmed the involvement of the immune system and suggested a different role of innate and adaptive immunity in the initiation and progression of HO [72].

Also mast cells are massively present in early lesions from FOP patients and in those observed in an injury-mediated mouse model [67,73]. These tissues expressed increased levels of Substance P, a known potent neuro-inflammatory factor, as a consequence of the dysregulated BMP pathway. Substance P was shown to be involved in injury-mediated ectopic bone formation and mast cell recruitment appeared to be an essential step of this process [74]. In accordance, pharmacological inhibition of mast cell activation by cromolyn treatment significantly reduced HO formation, providing preliminary observations about the importance of this pathway [73].

More recently, these data were confirmed in a physiologically relevant model of FOP, the conditional knock-in mouse expressing Acvr1^R206H^ [75]. Macrophages and mast cells obtained from early lesions of these mice were found to express increased levels of pro-inflammatory cytokines, and elevated and more prolonged response to LPS and Substance P treatment. Single depletion of mast cells or macrophages reduced the extent of ectopic ossification in these mice and the most consistent beneficial effect was obtained by the combined ablation of the two components [75].

In a single-case report, the long-term use of immunosuppressive drugs in an FOP patient with a graft-versus-host disease after a bone marrow transplantation due to aplastic anemia, apparently prevented the progression of the disease and flare-up occurrence. After immunosuppressant discontinuation, the disease returned in an active and severe phase. Although limited to a single patient, this case raised two different comments: pharmacological targeting of the immune system seemed to be useful in preventing FOP flare-ups; and that the presence of the wild-type immune system derived from a normal donor did not affect the course and the severity of the disease [76].

Recently, a comprehensive immunophenotyping of peripheral blood mononuclear cells (PBMCs) from a set of FOP patients was carried out [77]. Increased expression of specific adhesion molecules and cytokine receptors in monocytes such as CXCR4/CD184 and HLA-DR, and in particular of DNAM1/CD226, was observed in patients’ cells compared to control cells. If confirmed in other studies this could extend the cohort of investigated patients, suggesting the existence of a slightly activated state of these cells and would provide indications for new therapeutic targets [77].

Although many aspects regarding the immune-mediated component in FOP pathogenesis should be further investigated and clarified, all the evidences reported so far, support the idea that careful and specifically-addressed modulation of the immune system may provide beneficial effects in the management of FOP flare-ups, especially in the very early phase.

## 6. Targeting the Microenvironment of FOP Local Lesions

Besides triggers inducing the early inflammatory reaction, which are often not recognizable, the presence of an inductive microenvironment is certainly required to promote and sustain the whole process of ectopic ossification in FOP. The tissue metamorphosis leading to HO formation starts with a local strong inflammatory reaction and inflammation is a well-recognized cause of tissue hypoxia. A critical step in cell response to hypoxic conditions is the stabilization of the HIF1α transcription factor leading to the formation of heterodimers with the constitutively expressed HIF1β and resulting in the regulation of target gene expression [78].

Hypoxia can be a promoting factor of HO because it can favor/foster chondrogenesis. Indeed, the expansion and chondrogenic induction of mesenchymal precursors generally results in enhanced chondrogenic differentiation under hypoxia or low oxygen tension [79] and HIF1α signaling is critical during normal cartilage differentiation.

HIF1α was found to be highly expressed and active in three different mouse models of ectopic ossification: trauma-induced, genetic, and a hybrid model of genetic and trauma-induced HO. In these models, HIF1α induction resulted to be coincident with the expression of a master transcription factor of cartilage development, *Sox9* [80].

The presence of highly hypoxic conditions was confirmed by immunohistochemical analysis in early mouse and human FOP lesions [81]. BMP signaling of FOP stem cells from human exfoliated deciduous teeth (SHED) was found to be more prolonged and intense than in control cells, with enhanced chondrogenic properties under hypoxic conditions [81]. This phenomenon was linked to the downregulation of RABEP1/Rabaptin5, a modulator of the Rab5 GTPase, critical for endocytic trafficking operated by the HIF1β/HIF1β transcriptional complex. As a consequence BMP signaling is maintained through the retention of ACVR1 in the endosomal compartment [81]. In accordance, the extent of BMP activation was found to be significantly reduced by the genetic or pharmacological inhibition of HIF1α. Most importantly, both genetic loss in mesenchymal cells and pharmacologic inhibition of HIF1α by treatment with Apigenin, Imatinib, PX-478 and Rapamycin significantly impaired HO formation in vivo, by preventing mesenchymal condensation [80,81,82].

Among the inhibitors of hypoxia signaling, Imatinib is a tyrosine kinase inhibitor originally developed and successfully applied for chronic myeloid leukemia [83]. Based on the preclinical studies showing favorable biological properties on early stages of FOP lesions [80,81] and on its safety profile, Imatinib has been prescribed on an off-label basis in a non-trial setting in seven children showing continuous FOP flare-ups [84]. However, this study was not conclusive about the possible therapeutic utility of this treatment and underlined the need for controlled clinical trials to determine the efficacy of a treatment [84].

Concerning Rapamycin, which is a drug commonly used as an immunosuppressant, the inhibition of the accumulation of HIF1α is linked to the inhibition of the mTOR pathway operated by this compound. As already mentioned, mTOR signaling appeared to be up-regulated in cells expressing mutated *ACVR1* upon ActA induction and its inhibition by Rapamycin suppressed HO formation in vivo, in mice implanted with FOP-iPSCs cells together with a source of ActA [37].

Although these observations provided proof-of-principle for the effectiveness of Rapamycin in animal models of FOP, administration of this drug to two FOP patients failed to show convincing evidence of efficacy as a therapy for the management of the disease [38].

## 7. Concluding Remarks

**Figure 1:**
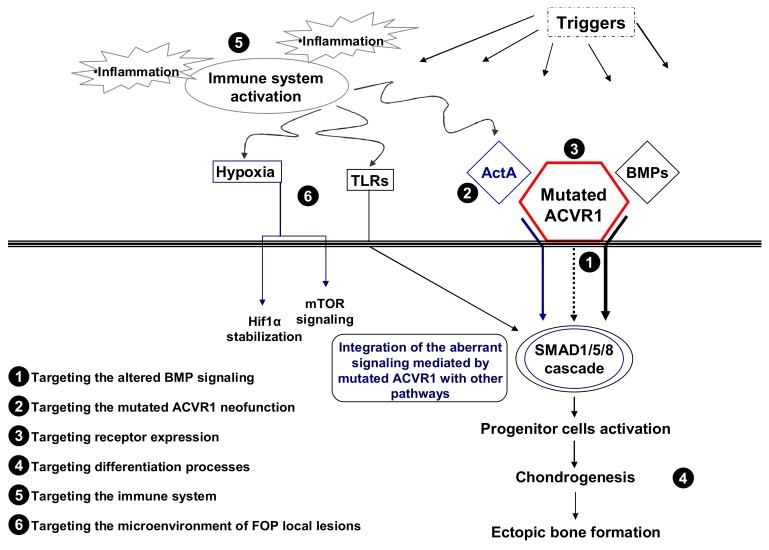
Schematic representation of molecular and cellular events involved in FOP pathogenesis. Druggable steps, some of which have recently been targeted to develop a treatment for the disease (see also Table 3), are indicated by numbers. TLRs, toll-like receptors; ActA, Activin A; BMPs, bone morphogenetic proteins.

FOP is a rare genetic disease and one of the most severe conditions of ectopic ossification in humans. So far there is no effective and specific treatment to cure this terrible disease. However, in the last ten years since the discovery of the causative gene, research on the pathogenic mechanisms underlying this condition has progressed impressively. Most importantly, it has provided several druggable targets to attack the disease by identifying the dysregulated pathways involved, their cross-talk with other signals, the key molecular and cellular processes that might create a favorable environment for the derailment of the tissue repair process leading to HO (Figure 1). This has allowed the multiplication of the efforts and the strategies to search for a therapy and for the first time we are close to the development of an etiological treatment for FOP. Moreover, Phase 2 and 3 trials are ongoing or ready to start (Table 2 and Table 3).

The episodic and unpredictable course of FOP makes management of the disease particularly challenging. The ideal treatment is a medication that could be easily administered to patients on a chronic basis to prevent acute phases and the creeping progression of the disease in the absence of evident symptoms with acceptable side-effects. This medication should be efficient in blocking lesion formation in a very early phase, thus strategies focused on targeting ligands or mutated receptor activity may be in principle more promising than others. However, due to the complexity of FOP pathogenesis to improve treatment tolerability and safety, the use of a combination of drugs with different targets that can act in synergy might be of help and could allow a reduction of the dosage required of each single treatment. Moreover, an effective therapy should prevent the ossification induced by surgical procedures to remove ectopic bone, thus improving the patients’ quality of life and mobility. Another critical aspect is represented by the possibility that patients may need to discontinue the medication for a number of reasons, in this case it is mandatory to be sure that no rebound effects will take place and an alternative therapy is available.

Despite the terrific advancements of the last years in FOP basic and translational research, some important elements of disease pathogenesis deserve further investigation, such as the role of the innate and adaptive immune system, the cross-talk among different transduction pathways, and the identification of biomarkers suitable to monitor disease and treatment efficacy. These studies would be of great help not only for a better comprehension of disease mechanisms, but also to provide new druggable targets and the possibility to expand the therapeutic options for FOP patients.

Besides FOP, understanding pathogenic mechanisms and identifying targets for therapy can benefit a large group of patients who suffer from other more common forms of HO that do not have a genetic cause.

The development of a therapy for rare diseases is a challenging process facing different and complicated issues such as finding the most effective cellular and molecular targets; the availability of preclinical models relevant for the study of disease pathophysiology; the ability to recruit a sufficient number of patients for clinical trials; and attracting the attention of pharmaceutical companies. In the complex world of research for therapeutic strategies for rare diseases, the support of associations of patients and families is particularly essential, to foster researcher networking and to stimulate the attention of pharmaceutical companies. From this point of view, the efforts made by the FOP patients associations represented by the International FOP Association (IFOPA) in the approaches to pharmaceutical companies is cited as an example and as a basis for guidelines for patient advocacy in the field of rare diseases [85].

## Figures and Tables

**Table 1:** *ACVR1* mutations associated with FOP.

Exon	Nucleotide *	Residue **	Domain
6	c.587T>C	p.Leu196Pro	GS
6	c.590-592delCTT	p.delPro197-Phe198insLeu	GS
6	c.605G>T	p.Arg202Ile	GS
6	c.617G>A	p.Arg206His	GS
6	c.619C>G	p.Gln207Glu	GS
7	c.774G>C/T	p.Arg258Ser	Kinase
7	c.774G>T	p.Arg258Ser	Kinase
8	c.974G>C	p.Gly325Ala	Kinase
8	c.982G>A/C/T	p.Gly328Arg/Trp	Kinase
8	c.983G>T/A	p.Gly328Val/Glu	Kinase
9	c.1067G>A	p.Gly356Asp	Kinase
9	c.1124G>C	p.Arg375Pro	Kinase

*ACVR1* gene consists of 11 exons, two 5′UTR exons and 9 protein coding exons, exon 6 corresponds to the fourth coding exon. * RefSeq NM_001111067.2; ** RefSeq NP_001104537.1.

**Table 2:** Approved clinical trials for investigational drugs to treat FOP.

Drug	Company	Trial Phase	Title *	ClinicalTrials.gov Identifier *
Palovarotene	Clementia	Phase3 (pivotal)	An Efficacy and Safety Study of Palovarotene for the Treatment of FOP	NCT03312634
REGN2477	Regeneron	Phase 2	A Study to Examine the Safety, Tolerability and Effects on Abnormal Bone Formation of REGN2477 in Patients With Fibrodysplasia Ossificans Progressiva (LUMINA-1)	NCT03188666

* From the ClinicalTrials.gov: https://clinicaltrials.gov/ct2/results?cond=FOP&term=&cntry=&state=&city=&dist= accessed on 21 February 2018. A Natural History Study of Fibrodysplasia Ossificans Progressiva (FOP) (ClinicalTrials.gov Identifier: NCT02322255), is also ongoing to define disease progression, clinical features, impact of patients’ physical functioning, provide information useful for interventional trials.

**Table 3:** Druggable targets and recent approaches to develop a treatment for FOP.

Identification of Sensitive Targets		Strategy	Comments
Targeting the altered signaling	Dysregulated BMP signaling	Development of pharmacological inhibitors of the kinase function	In vitro evidences; preclinical mouse models (Dorsomorphin, LDN-189193; LDN LDN-212854 other derivatives, …) Possible application of kinase inhibitors developed for other conditions?
		Screening of FDA-approved compounds able to interfere with the dysregulated BMP signaling	In vitro evidences; preclinical mouse models. Perhexiline tested in patients on a off-label basis in a non-trial setting
	Neofunction of the mutated receptor (responsiveness to ActA)	Development of blocking anti-ActA antibodies	In vitro evidences; preclinical FOP mouse models; REGN2477 recruiting Phase 2 trial
		Screening of FDA-approved compounds able to interfere with the ActA/mutated ACVR1 signaling	In vitro evidences; preclinical mouse models (mTOR inhibitors, Rapamycin)
Targeting differentiation processes	Chondrogenesis as a critical differentiation step in HO formation	Maintenance of the retinoid signaling active to block chondrogenic differentiation by using RARγ agonists	In vitro evidences; preclinical FOP mouse models. Palovarotene on Phase 3 trial
Targeting the expression of the ACVR1/Alk-2 receptor	Transcriptional level	Screening of molecules potentially able to down-modulate expression of the *ACVR1* gene at transcriptional level	In vitro evidences; preclinical HO mouse models (Dipyridamole)
	Post-transcriptional level	*ACVR1* targeting by microRNA	Proof of principle
		Development of Anti-sense oligonucleotide (AON) to promote exon skipping and *ACVR1* mRNA targeting to the non-sense mediated degradation pathway	
		Development of allele-specific RNAi molecules (ASP-RNAi) able to target the expression of mutant *ACVR1* alleles	
Targeting the immune system	Modulation of the immune response & inflammation	Targeted ablation of macrophages and mast cells	In vitro evidences; preclinical HO/FOP mouse models
		Use of corticosteroids or immunosuppressant drugs	Corticosteroids are currently in use to manage FOP flare-ups; single case report of long term use of immunosuppressant is reported
Targeting the microenvironment of FOP local lesions	Modulation of hypoxia	Pharmacological inhibition of HIF-1α pathway (Apigenin, Imatinib, PX-478 and Rapamycin)	In vitro evidences; preclinical mouse models. Imatinib and Rapamycin tested in patients on a off-label basis in a non-trial setting
